# Valproic acid and butyrate induce apoptosis in human cancer cells through inhibition of gene expression of Akt/protein kinase B

**DOI:** 10.1186/1476-4598-5-71

**Published:** 2006-12-11

**Authors:** Jihong Chen, Feras M Ghazawi, Wafae Bakkar, Qiao Li

**Affiliations:** 1 Department of Pathology and Laboratory Medicine, Faculty of Medicine, University of Ottawa, 451 Smyth Road, Room 4155, Ottawa, Ontario, K1H 8M5, Canada

## Abstract

**Background:**

In eukaryotic cells, the genomic DNA is packed with histones to form the nucleosome and chromatin structure. Reversible acetylation of the histone tails plays an important role in the control of specific gene expression. Mounting evidence has established that histone deacetylase inhibitors selectively induce cellular differentiation, growth arrest and apoptosis in variety of cancer cells, making them a promising class of anticancer drugs. However, the molecular mechanisms of the anti-cancer effects of these inhibitors have yet to be understood.

**Results:**

Here, we report that a key determinant for the susceptibility of cancer cells to histone deacetylase inhibitors is their ability to maintain cellular Akt activity in response to the treatment. Also known as protein kinase B, Akt is an essential pro-survival factor in cell proliferation and is often deregulated during tumorigenesis. We show that histone deacetylase inhibitors, such as valproic acid and butyrate, impede Akt1 and Akt2 expression, which leads to Akt deactivation and apoptotic cell death. In addition, valproic acid and butyrate induce apoptosis through the caspase-dependent pathway. The activity of caspase-9 is robustly activated upon valproic acid or butyrate treatment. Constitutively active Akt is able to block the caspase activation and rescues cells from butyrate-induced apoptotic cell death.

**Conclusion:**

Our study demonstrates that although the primary target of histone deacetylase inhibitors is transcription, it is the capacity of cells to maintain cellular survival networks that determines their fate of survival.

## Background

In eukaryotic cells, histones play an important role in transcription control by packing the genomic DNA into an array of nucleosomes and higher order chromatin structure [1-3]. The amino-terminal tails of histones are rich in positively charged lysine residues which form tight contacts with the negatively charged DNA backbones, restricting the accessibility of DNA for the binding of transcription regulators [4,5]. This restrictive milieu needs to be relieved to allow the regulation of chromatin structure and function, and ultimately for gene activation to occur, which often is achieved through acetylation of the histone tails by histone acetyltransferases [6-9]. On the other hand, deacetylation of the histone tails by histone deacetylases (HDAC) restores the histone restriction, resulting in gene repression.

Malignant cells gain various phenotypic characteristics during the development of cancer, which permit them to proliferate abnormally and eventually invade surrounding tissues. Numerous studies have demonstrated the importance of epigenetic alteration in cancer onset. This has raised the possibility of controlling transcription as a potential approach in cancer therapeutics [10]. Valproic acid, a short-chain fatty acid, is a well established drug in the treatment of epilepsy, migraine, cluster headaches and for the control of a variety of seizures [11]. Butyrate, also a short chain fatty acid, naturally produced by bacterial fermentation in the colon, has been designated as the most potent fatty acid in arresting cell proliferation [12]. Both compounds are classified as HDAC inhibitors [13,14]. Many studies have shown that HDAC inhibitors selectively induce cellular differentiation, growth arrest and apoptosis in cancer cells, making these inhibitors a promising new class of anticancer drugs [15-17]. However, the molecular mechanisms of the anticancer effects of the HDAC inhibitors have yet to be understood.

Akt, also known as protein kinase B, is a serine/threonine protein kinase and a key player in the regulation of apoptosis, proliferation and tumorigenesis [18-20]. Currently, three mammalian isoforms have been identified, namely Akt1/PKBα, Akt2/PKBβ and Akt3/PKBγ [21]. Akt1 is the predominant isoform in most tissues while Akt2 is highly expressed in skeletal muscle, heart, liver and kidney [22,23]. Akt3 exhibits a more restricted pattern of distribution, mostly found in testis and brain [24]. The phosphorylation of a conserved threonine residue (Thr^308 ^in Akt1, Thr^309 ^in Akt2 and Thr^305 ^in Akt3), upon growth factor stimulation, is required for Akt activation, while the phosphorylation of a serine residue (Ser^473 ^in Akt1, Ser^474 ^in Akt2 and Ser^474 ^in Akt3) is only required for maximal Akt activity [21,24-26]. Constitutively active Akt can block apoptosis induced by several diverse treatments, such as growth factor withdrawal, UV irradiation, matrix detachment and DNA damage [27-29].

Akt promotes cellular survival by directly phosphorylating transcription factors that control the expression of pro-survival or anti-apoptotic genes. For instance, the forkhead family of transcription factors resides in the nucleus where they induce the expression of pro-apoptotic genes [30]. However, in the presence of active Akt, these transcription factors are exported out of the nucleus and sequestered in the cytoplasm by 14-3-3 proteins [25]. Cytoplasmic retention of these transcription factors acts as a negative regulation of apoptotic machinery. In contrast, NF-kB, a transcription factor involved in promoting cell survival, is positively influenced by Akt that phosphorylates and activates IKKα, leading to the destruction of I-kB and the entry of NF-kB into the nucleus [31-33].

Direct phosphorylation of proteins involved in apoptosis is another mechanism by which Akt promotes cellular survival. BAD, a member of the Bcl-2 family is sequestered by 14-3-3 proteins in the cytosol upon phosphorylation by Akt [34]. This event prevents BAD from interacting with pro-survival members of the Bcl-2 family at the mitochondrial membrane [24,25]. Akt prevents cytochrome release by maintaining structural integrity of the mitochondria and is also involved in post-mitochondrial events through phosphorylation of caspase-9 [24,35-37]. Recent studies demonstrated that Akt positively regulates the protein stability and transcriptional activity of coactivator p300 [38,39]. Since p300 contains an intrinsic histone acetyltransferase activity, it is possible that Akt also promotes cellular survival through the regulation of chromatin dynamics.

HDAC inhibitors have a range of anti-cancer activities including the induction of apoptosis in transformed culture cells and in cancers. Some studies have observed a decreased Akt activity following treatment with HDAC inhibitors. Here we report that the induction of cancer cell apoptosis by HDAC inhibitors, such as valproic acid and butyrate, is achieved through inhibition of gene expression of Akt1 and Akt2, pro-survival factors involved in many cell signalling pathways. In addition, valproic acid and butyrate induce apoptotic cell death through the caspase-dependent pathway.

## Results

### Valproic acid and butyrate repress Akt1 and Akt2 expression and induce apoptosis in HeLa cells

**Figure 1:**
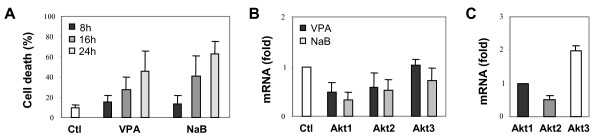
Valproic acid and butyrate repress Akt expression and induce apoptosis in HeLa cells. (A) Cell death was measured by flow cytometry analysis of subG1 population following propidium iodide staining of permibilized HeLa cells after exposure to valproic acid (VPA, 2 mM) or sodium butyrate (NaB, 5 mM) for 8, 16 or 24 hours. (B) Following 16 hours of valproic acid or butyrate treatment, the mRNA levels of Akt1, Akt2 and Akt3 in the HeLa cells were determined by quantitative real-time RT-PCR analysis with a TaqMan probe protocol. 18S rRNA was used as an internal control. Results show fold variations of treated cells in comparison to untreated controls. Error bars represent standard deviations of three independent experiments. (C) The relative mRNA abundance of Akt1, Akt2 and Akt3 isoforms of the HeLa cells was assessed by quantitative RT-PCR and plotted as fold variations of Akt1. Error bars represent the standard deviations of three independent experiments.

HDAC inhibitor-induced growth arrest and apoptotic cell death have been observed in a variety of solid and hematological cancers, but the mechanisms of their action remain obscure [17]. To understand the mechanism of apoptotic cell death induced by HDAC inhibitors such as valproic acid and butyrate, we employed HeLa cells derived from a human cervical cancer [40]. As shown in Fig. 1A, butyrate induced significant HeLa cell death after 16 hours of treatment (about 41%, P < 0.05) and more significantly after 24 hours (about 63%, P < 0.005) as determined by flow cytometry analysis of the subG1 populations. Valproic acid was also able to induce significant HeLa cell death after 24 hours of treatment (Fig. 1A), but in a lesser degree (about 46%, P < 0.05).

Affymetrix microarray analysis of gene expression profiles revealed that butyrate treatment down-regulated pro-apoptotic BAD, BAX and BAK, genes associated with the mitochondrial pathway, while genes associated with the death receptor pathway, such as Fas, FasL, Trail and DR5, were basically not affected by the treatment (data not shown). However, Akt mRNA was reduced in the HeLa cells following butyrate treatment (data not shown). Since Akt is a key regulator of cellular survival, we studied the effects of HDAC inhibitors on Akt expression by using quantitative real-time RT-PCR analysis with specific primers and corresponding TaqMan probes for different Akt isoforms. As shown in Fig. 1B, following 16 hours of butyrate treatment, the levels of Akt1 and Akt2 mRNA were reduced by about 70% and 50% respectively as compared to untreated control cells. Valproic acid treatment also reduced the levels of the Akt1 and Akt2 mRNA, but to a lesser degree (Fig. 1B). Intriguingly, the level of Akt3 mRNA was basically unaffected by valproic acid or butyrate treatment (Fig. 1B). Quantification of the relative mRNA levels of different Akt isoforms showed a distinct expression profile in the HeLa cervical cancer cells. Akt3 is the most abundant of Akt isoforms, about 2 and 4 fold more than Akt1 and Akt2 respectively (Fig. 1C), which is not a normal Akt isoform expression pattern for cervical epithelial cells.

**Figure 2:**
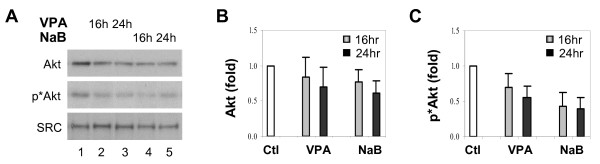
Deactivation of Akt by valproic acid and butyrate. (A) Equal amounts of whole cell extract (50 μg) were used to examine the levels of endogenous Akt and phospho-Akt in the HeLa cells treated with valproic acid (VPA, 2 mM) or sodium butyrate (NaB, 5 mM) for 16 or 24 hours. The blot was then stripped and reprobed with a SRC antibody for protein loading controls. (B) Quantitative analysis of the Akt blots is expressed as fold variations compared to untreated control after being normalized to the loading controls. Error bars represent standard deviations of three independent experiments. (C) The quantification of phospho-Akt Western blots was performed as described for panel B.

Next, we examined whether the abundance of total Akt protein is affected by valproic acid or butyrate treatment. As shown in Fig. 2A, a decline in the abundance of Akt protein was apparent following 16 hours of valproic acid or butyrate treatment, which became more evident after 24 hours of treatment. On the other hand, the levels of SRC protein were not affected by the treatments (Fig. 2A) as previously reported [41]. Quantitative Western blot analysis revealed that cells treated with butyrate exhibit a more significant decrease in Akt protein than the valproic acid treated cells; nearly 40% of Akt reduction was observed after 24 hours of butyrate treatment compared to 30% reduction after valproic acid treatment (Fig. 2A and 2B). We also assessed the phosphorylation status of Akt since its activity is regulated by phosphorylation [20,25]. Western blot analysis using an antibody specific for phosphorylated Ser^473 ^residue in Akt demonstrated that the phosphorylation level of Akt protein started to decline after 16 hours of butyrate treatment, and again valproic acid had a weaker effect (Fig. 2A). Quantification of the Western blots verified that the levels of Akt phosphorylation were reduced by about 60% and 40% after 24 hours of butyrate and valproic acid treatment respectively, as compared to untreated control cells (Fig. 2C). Taken together, these data suggest that the down-regulation of Akt activity by HDAC inhibitors, such as valproic acid and butyrate, is achieved through inhibition of gene expression of Akt1 and Akt2.

### Valproic acid and butyrate induce caspase-3 activation

**Figure 3:**
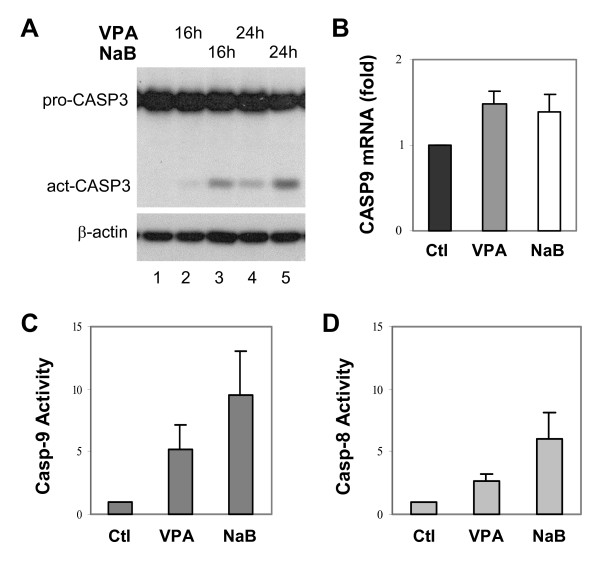
Valproic acid and butyrate induce caspase activation. (A) Equal amounts of whole cell extracts (50 μg) were used for Western blot analysis of caspase-3 in HeLa cells upon treatment with valproic acid (VPA, 2 mM) or sodium butyrate (NaB, 5 mM) for 16 or 24 hours. The blots were then stripped and reprobed with a β-actin antibody for protein loading controls. (B) The level of caspase-9 mRNA in the HeLa cells was examined by quantitative real-time RT-PCR analysis following valproic acid or butyrate treatment. Error bars represent standard deviations of three independent experiments. (C) and (D) Following 16 hours of valproic acid or butyrate treatment, the HeLa cells were harvested and assayed for caspase-9 and caspase-8 activities in parallel. Results show fold induction of the activities relative to untreated controls after background subtraction of zero time signals. Error bars represent the standard deviations of five independent experiments.

Programmed cell death can be mediated through either caspase-dependent or -independent mechanisms. To determine the molecular pathway of cell apoptotic death induced by HDAC inhibitors, such as valproic acid and butyrate, we examined the active status of caspase-3 following the treatment of HeLa cells. Western blot analysis with an antibody specifically against caspase-3 showed that the HeLa cells contain an abundance of the precursor caspase-3 (Fig. 3A). However, the cleaved or activated form of caspase-3 was only observed following valproic acid or butyrate treatment (Fig. 3A). Again, butyrate had a stronger effect than valproic acid on inducing caspase-3 activity, correlating directly with its higher efficacy than valproic acid on the deactivation of Akt and on the induction of cell apoptotic death (Fig. 1 and 2). Taken together, these data suggest that apoptotic cell death induced by HDAC inhibitors, such as valproic acid and butyrate, is mediated through the caspase-dependent pathway.

### Valproic acid and butyrate activate both the caspase-8 and caspase-9

Caspase-3 is an executor caspase that can be cleaved or activated by either caspase-9, the initiator caspase of the mitochondrial pathway, or caspase-8, the initiator caspase of the death receptor pathway. Affymetrix microarray analysis showed that butyrate treatment increased the level of caspase-9 mRNA to a small extent (data not shown). Quantitative real-time RT-PCR analysis confirmed that the capase-9 mRNA of the HeLa cells was indeed increased to about 1.5 fold upon valproic acid or butyrate treatment (Fig. 3B). Next, we assessed the enzymatic activity of caspase-9 by using a fluorescence based assay in the presence or absence of LEHD-CHO, a specific inhibitor for the caspase-9. As shown in Fig. 3C, valproic acid or butyrate treatment induced the caspase-9 activity and again butyrate induced activity of the caspase-9 more robustly than valproic acid, about 9 fold versus 4 fold.

Caspase-8 can be activated by HDAC inhibitors through the death receptors in several cancer cell lines [42-44]. To determine the role of the death receptor pathway in HDAC inhibitor induced HeLa cell death, we evaluated the activity of caspase-8 in the HeLa cells by using a fluorescence based assay. Following treatment with valproic acid or butyrate, the activity of caspase-8 was increased to about 2 and 5 fold respectively, but to a lesser degree in comparison to the caspase-9 activity (Fig. 3D). Taken together, our data suggest that the HDAC inhibitor-induced apoptotic cell death is attained by activating both the caspase-8 and caspase-9 activities.

### Introduction of constitutively active Akt prevents butyrate-induced apoptosis

**Figure 4:**
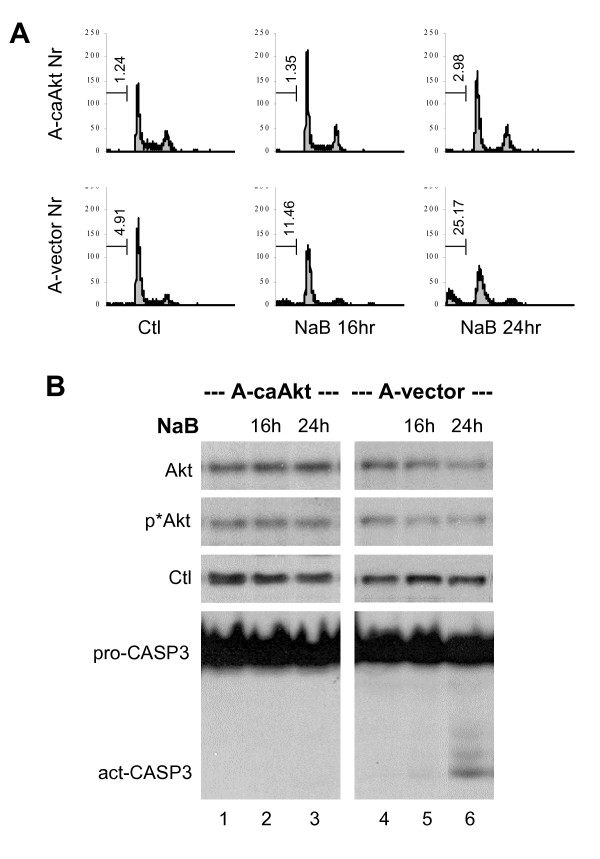
Constitutively active Akt counteracts butyrate-induced apoptosis. (A) Ovarian carcinoma A2780S cell lines stably integrated with an expression plasmid for constitutively active Akt (A-caAkt) or an empty vector (A-vector) were used for flow cytometry analysis of cells stained with propidium iodide after 16 or 24 hours exposure to sodium butyrate (NaB, 5 mM). The percentage of cells in the subG1 population is indicated in the corresponding graphs. Untreated cells were used as controls. (B) Equal amounts of whole cell extracts (50 μg) were used for Western blot analysis of Akt, phospho-Akt and casepase-3 levels in the A-caAkt and A-vector cells following butyrate treatments. The blot was then stripped and reprobed for loading control (Ctl) with a SRC antibody.

To further determine the role of Akt in counteracting the apoptosis induced by HDAC inhibitors, we employed an ovarian cancer cell line stably integrated with an expression plasmid for a constitutively active Akt and a control cell line stably integrated with an empty vector [38]. Flow cytometry analysis revealed that butyrate was able to induce significant apoptotic death in the control cells but not in the cells expressing the constitutively active Akt (Fig. 4A). Western blotting analysis demonstrated that the levels of Akt protein and phosphorylated Akt were diminished by the treatment in the control cells, but not in the cells expressing the constitutively active Akt (Fig. 4B). In addition, following butyrate treatment the cleaved or activated form of caspase-3 was only observed in the control cells but not in the cells expressing the constitutively active Akt (Fig. 4B). Taken together, these data strongly support the notion that the effect of butyrate on cellular survival is determined by the cellular Akt activity of the cells.

### Valproic acid and butyrate do not affect the Akt activity and cellular survival of SiHa cells

**Figure 5:**
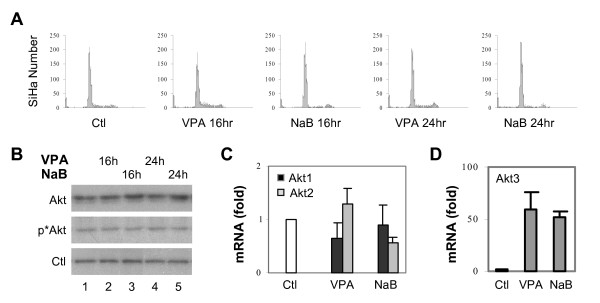
SiHa cell survival is not affected by valproic acid and butyrate. (A) Flow cytometry analysis of propidium iodide uptake of the SiHa cells following exposure to valproic acid (VPA, 2 mM) or sodium butyrate (NaB, 5 mM). Untreated SiHa cells were used as control and the time of treatments was for 16 or 24 hours. (B) Equal amounts of whole cell extracts (50 μg) were used for Western blot analysis of endogenous Akt and phospho-Akt of the SiHa cells following treatment with valproic acid or butyrate for 16 or 24 hours. The blot was then stripped and reprobed for loading control (Ctl) with a SRC antibody. (C) and (D) Following 16 hours of valproic acid or butyrate treatment, the mRNA levels of Akt1, Akt2 and Akt3 in the SiHa cells were examined by quantitative real-time RT-PCR analysis. 18S was used as an internal control. Results show fold variations of treated cells compared to untreated controls. Error bars represent standard deviations of three independent experiments.

To determine further the significance of Akt activity in apoptotic cell death induced by HDAC inhibitors such as valproic acid or butyrate, we screened several cancer cell lines for their Akt activity and viability in response to the treatments, and found strong correlation between the ability of cells to maintain their Akt activity and to survive valproic acid or butyrate treatment. One of the cell lines is SiHa, derived from a human cervical cancer like HeLa cells [45]. As shown in Fig. 5A, valproic acid or butyrate treatment did not affect the cellular survival of the SiHa cells as assessed by flow cytometry analysis. When Western blot analysis of Akt protein was performed, we observed a moderate increase in Akt protein in the SiHa cells following valproic acid and butyrate treatment, rather than a decrease as in the HeLa cells (Fig. 5B). In addition, the phosphorylation status of Akt in the SiHa cells was not reduced by the treatments (Fig. 5B). Next, we performed quantitative real-time RT-PCR analysis to assess the mRNA levels of Akt isoforms. As shown in Fig. 5C, both valproic acid and butyrate decreased the levels of Akt1 and Akt2 mRNA to a certain degree, but not significantly. In contrast, the treatments increased the levels of Akt3 mRNA by over 50 fold in comparison to untreated control (Fig. 5D), which is consistent with the augmented levels of Akt protein as assessed by the Western blot analysis (Fig. 5B).

**Figure 6:**
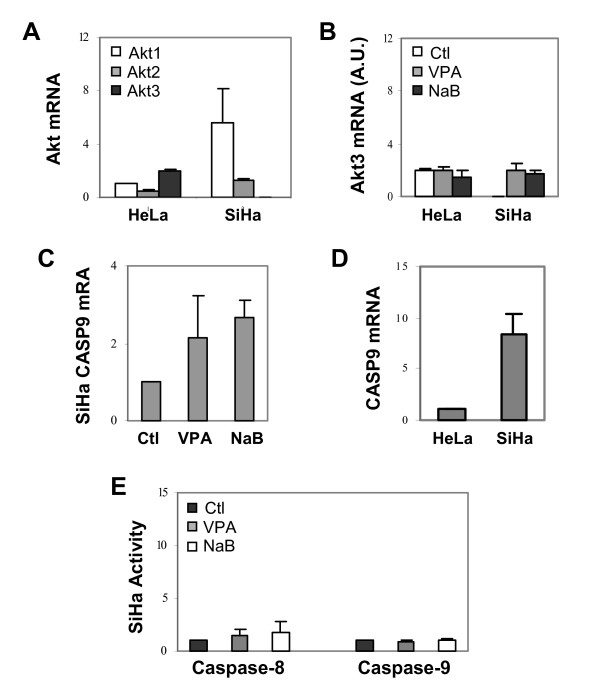
The Akt mRNA levels and caspase activities of the SiHa cells. (A) The relative mRNA abundance of Akt1, Akt2 and Akt3 in the HeLa and SiHa cells were assessed by quantitative RT-PCR and plotted as fold variations of Akt1 mRNA level of the HeLa cells. 18S was used as an internal control. Error bars represent standard deviations of three independent experiments. (B) The relative level of Akt3 mRNA following 16 hours of exposure to valproic acid (VPA, 2 mM) or sodium butyrate (NaB, 5 mM) was plotted as arbitrary units (A.U.) of the mRNA abundance. (C) The level of caspase-9 mRNA in the SiHa cells was examined by quantitative real-time RT-PCR analysis following valproic acid or butyrate treatment. Results are expressed as fold induction of the transcripts relative to untreated controls. Error bars represent standard deviations of three independent experiments. (D) The experimental set up was as in panel A except that the caspase-9 mRNA level in untreated SiHa cells was compared to untreated HeLa cells. (E) Following 16 hours of valproic acid or butyrate treatment, the SiHa cells were assayed for their caspase-8 and caspase-9 activities. Results show fold induction of the activities relative to untreated controls after background subtraction of zero time signals. Error bars represent standard deviations of five independent experiments.

Quantification of the relative levels of Akt isoforms revealed a very different expression profile between the HeLa and SiHa cells. Akt3 was the most abundant of Akt isoforms in the HeLa cells (Fig. 6A). In contrast, the SiHa cells contained an extremely low level of Akt3 mRNA under normal growth conditions, whereas Akt1 mRNA was the most abundant, about 4 fold more than Akt2 (Fig. 6B). The levels of total Akt mRNA and protein in the SiHa cells were about 2 fold higher than those of HeLa cells (Fig. 6A and data not shown). Intriguingly, treatment of the SiHa cells with valproic acid or butyrate only increased the level of Akt3 mRNA to that of untreated HeLa cells (Fig. 6B). In addition, the level of caspase-9 mRNA was also increased in the SiHa cells and the total caspase-9 mRNA of the SiHa cells was about 9 fold higher than that of the HeLa cells (Fig. 6C and 6D). Therefore, we assessed the enzymatic activity of caspase-9 by using a fluorescence based assay. As shown in Fig. 6E, valproic acid or butyrate treatment failed to induce the activity of caspase-9 in the SiHa cells. However, the treatments resulted in a slight increase of the caspase-8 activity but no caspase-3 activation (Fig. 6E and data not shown). Taken together, these data indicate that the ability of cells to survive valproic acid or butyrate treatment depends on their ability to maintain the cellular activity of Akt.

## Discussion

HDAC inhibitors, inducers of differentiation or apoptosis of some cervical and ovarian cancer cells, have become a new class of drugs for treatment of a variety of cancers [17]. However, many questions concerning their mechanisms of action and their therapeutic potentials for different cancers are largely unanswered. These are intimate related issues due to the heterogeneity of the genetic lesion and epigenetic alteration of the cancer. The molecular mechanisms of HDAC inhibitors as cancer therapeutics may be highly dependent on the type or cause of the cancer. Our study demonstrated that HDAC inhibitors, such as valproic acid and butyrate, induce apoptosis in HeLa cervical cancer cells by inhibition of gene expression of Akt1 and Akt2. In addition, the apoptotic cell death induced by valproic acid or butyrate is mediated through the caspase-dependent pathways.

Inhibition of histone deacetylation and alteration of chromatin structure often lead to transcriptional activation. Numerous studies have shown that, through its chromatin remodeling activities, HDAC inhibitors are capable of modulating gene transcription involved in various cellular processes such as cell cycle progression, differentiation and apoptosis [15]. Gene silencing or abnormal expression is a hallmark of many forms of malignancy. The efficacy of HDAC inhibitors in cancer therapeutics may well come from restoring silenced gene expression since transcription is the primary target of HDAC inhibitors. Increasing the expression of some pro-apoptotic proteins, such as TRAIL and p21, has been shown to be one of the molecular mechanisms by which the HDAC inhibitors induce cancer cell death [46,47]. However, HDAC inhibitor treatment can also result in gene repression [48]. Our study demonstrates that indeed this may be an alternative mechanism by which HDAC inhibitors induce cell apoptotic death. Treatment of HeLa cells with HDAC inhibitors such as valproic acid and butyrate leads to inhibition of Akt1 and Akt2 expression, consequently to deactivation of cellular Akt (Fig. 1 and 2).

The link between the action of HDAC inhibitors and the down-regulation of Akt phosphorylation has been reported previously [18,49,50]. In both 87MG and PC3 cells, treatment with HDAC inhibitors decreases the levels of Akt phosphorylation [49]. We, for the first time, show that the down-regulation of Akt activity is a consequence of inhibition of Akt1 and Akt2 expression by valproic acid and butyrate. It appears that these inhibitors repress Akt1 and Akt2 transcription thereby depleting the cellular Akt protein. The negative effect of valproic acid or butyrate on Akt expression could be mediated through a direct or indirect mechanism, i.e. through inhibiting deacetylation of nonhistone proteins, such as transcription regulators, or gene activation of some negative regulators of Akt gene transcription. Understanding the molecular basis by which Akt gene expression is regulated will ultimately help us to design better strategies to treat cancer.

The molecular basis for the action of HDAC inhibitors in caner therapeutics is one of the important questions remaining to be answered. If transcription is the primary target of HDAC inhibitors, then normal cells should be equally if not more susceptible toward the HDAC inhibitors. Our study demonstrated that the key answer may reside in the competency of signaling pathways to maintain the cellular activity of Akt in response to the treatment. Being a key pro-survival factor, Akt is often deregulated during tumorigenesis [19]. For example, HeLa cells exhibit an abnormal expression pattern of Akt isoforms, in which Akt3 is the most abundant of Akt isoforms (Fig. 1C). This deregulation of Akt may sensitize cancer cells to HDAC inhibitors. Without Akt to mediate its pro-survival activity, the cells may be more prone to apoptosis.

The role of caspase in HDAC inhibitor-induced apoptosis has been controversial or may be dependent on cellular context. Some studies have established clear roles for the death receptors and caspase-8 in this process [42,43,47,51]. Our study demonstrated that valproic acid and butyrate promote apoptosis in HeLa cervical cancer cells through a caspase-dependent mechanism (Fig. 3). Most intriguingly, the caspase-9 activity is more robustly activated than caspase-8 upon the valproic acid or butyrate treatment (Fig. 3). In addition, introduction of constitutively active Akt blocks caspase-3 activation and rescues cells from apoptotic death upon butyrate treatment (Fig. 4). Akt directly regulates caspase-9 activity and promotes cellular survival at post-mitochondrial level [35,36]. It is likely that deactivation of Akt plays a primary role in the activation of caspase-9 during the HeLa apoptotic death induced by valproic acid or butyrate.

Apparently, apoptosis induced by HDAC inhibitors is associated with various disarrays of different cell signaling pathways. The precise molecular mechanisms involved in the cancer therapeutics of HDAC inhibitors may depend highly on the cellular context or the genetic lesion and epigenetic background of the cancer. For targeted or customized cancer therapy, it is essential to understand the distinct mechanisms of apoptotic cell death induced by HDAC inhibitors. Our study demonstrates that although the primary target of HDAC inhibitors may be transcription, it is the cellular environment, or the ability of cells to maintain their survival protein networks that determines their fate, to die or to survive in response to the treatment.

## Methods

### Cell culture and reagents

The cells were maintained in Dulbecco's Modified Eagle Medium supplemented with 10% fetal bovine serum (Invitrogen) at 37°C with 5% CO_2_. Valproic acid and sodium butyrate were purchased from Sigma. Antibodies against Akt, phosphor-Akt and caspase3 were obtained from Cell Signalling.

### Flow cytometry analysis

Following exposure to valproic acid (2 mM) or sodium butyrate (5 mM) for 8, 16 or 24 hours, cells were detached from tissue culture dishes by trypsinization, combined with floating cells and fixed in 70% ethanol for 30 minutes at -20°C. After washing with phosphate-buffered saline, the cells were incubated with 50 μg/ml of RNase A and 50 μg/ml of propidium iodide for 30 minutes at 37°C. DNA contents of the cells were then profiled by fluorescence activated cell analyzer (Beckman Coulter) to determine the distribution of cells in different phases of the cell cycle [38].

### Quantitative real-time RT-PCR

Total RNA was isolated using RNeasy Mini Kit (Qiagen) and reverse transcribed using random primers and Superscript II (Invitrogen). Serial dilution of the cDNA was used to determine the appropriate concentration required for the real-time PCR amplification which was carried out by using TaqMan reporter assay with a 7500 Fast Real-Time PCR System (Applied Biosystems). Gene specific primers and TaqMan probes used for the amplification were ordered from Applied Biosystems. Quantification of mRNA levels was performed by using 18S rRNA as an internal control.

### Protein extraction and Western blot analysis

Following various treatments, cells were washed and harvested. The cell pellets were suspended and incubated for 30 minutes at 4°C in whole-cell extraction buffer consisting of 10% glycerol, 50 mM Tris-HCl (pH 7.6), 400 mM NaCl, 1 mM dithiothreitol, 1 mM phenylmethylsulfonyl fluoride, and 1% Nonidet P-40. The lysates were then centrifuged at 14,000 g for 10 minutes at 4°C. Protein concentrations were determined by Bradford assay (Bio-Rad) using bovine serum albumin as standard. PerkinElmer Life Sciences ECL system was used for the detection and Scion Image software was used to quantify the Western blots [41].

### Caspase activity assay

Following various treatments, cells were assayed for caspase activities by using fluorescent assay kits (Clontech) and a microplate fluorometer (Molecular Devices). The caspase-8 and caspase-9 activities were determined with the same batch of lysates simultaneously and were measured from zero time to 2 hours as the manufacturer recommended. The specificity of the fluorescent signal was determined by IETD-fmk and LEHD-CHO, the specific inhibitors for the caspase-8 and caspase-9 respectively.

## Declaration of Competing Interests

The author(s) declare that they have no competing interests.

## Authors' contributions

JC carried out flow cytometry, Western and caspase analysis; FMG participated in real-time RT-PCR analysis; WB participated in Western and real-time RT-PCR analysis; QL designed and supervised the study, and helped to draft the manuscript. All authors read and approved the final manuscript.
